# Parasitism Performance and Fitness of *Cotesia vestalis* (Hymenoptera: Braconidae) Infected with *Nosema* sp. (Microsporidia: Nosematidae): Implications in Integrated Pest Management Strategy

**DOI:** 10.1371/journal.pone.0100671

**Published:** 2014-06-26

**Authors:** Nadia Kermani, Zainal-Abidin Abu Hassan, Amalina Suhaimi, Ismail Abuzid, Noor Farehan Ismail, Mansour Attia, Idris Abd Ghani

**Affiliations:** 1 School of Environmental and Natural Resource Sciences, University National Malaysia, Bangi, Malaysia; 2 Faculty of Medicine, University Technology MARA UITM, Shah Alam, Malaysia; French National Institute for Agricultural Research (INRA), France

## Abstract

The diamondback moth (DBM) *Plutella xylostella* (L.) has traditionally been managed using synthetic insecticides. However, the increasing resistance of DBM to insecticides offers an impetus to practice integrated pest management (IPM) strategies by exploiting its natural enemies such as pathogens, parasitoids, and predators. Nevertheless, the interactions between pathogens and parasitoids and/or predators might affect the effectiveness of the parasitoids in regulating the host population. Thus, the parasitism rate of *Nosema*-infected DBM by *Cotesia vestalis* (Haliday) (Hym., Braconidae) can be negatively influenced by such interactions. In this study, we investigated the effects of *Nosema* infection in DBM on the parasitism performance of *C. vestalis*. The results of no-choice test showed that *C. vestalis* had a higher parasitism rate on non-infected host larvae than on *Nosema*-treated host larvae. The *C. vestalis* individuals that emerged from *Nosema*-infected DBM (F1) and their progeny (F2) had smaller pupae, a decreased rate of emergence, lowered fecundity, and a prolonged development period compared to those of the control group. DBM infection by *Nosema* sp. also negatively affected the morphometrics of *C. vestalis*. The eggs of female *C. vestalis* that developed in *Nosema*-infected DBM were larger than those of females that developed in non-infected DBM. These detrimental effects on the F1 and F2 generations of *C. vestalis* might severely impact the effectiveness of combining pathogens and parasitoids as parts of an IPM strategy for DBM control.

## Introduction

The diamondback moth (DBM) *Plutella xylostella* (L.) (Lep., Plutellidae) is a cosmopolitan pest that causes serious damage to a wide variety of cruciferous and other crops. This pest has been routinely controlled using chemical insecticides; however, the excessive use of these products has caused several concerns related to the development of resistance [1]–[4], the presence of pesticide residues in the environment and human food [1], and the impact of pesticide applications on populations of non-target organisms [5], [6]. Microsporidia have long been considered an attractive alternative to synthetic chemical insecticides for pest management because of their significant environmental and economic advantages over chemical insecticides. They are environmentally safe, acceptable, cause no pollution and are also considered important regulators of population dynamics [7]. Recently, many investigations have been focusing on the use of microsporidia as biological control agents of some economically important insect pests [8]. For example, *Paranosema locustae* (*Nosema*) was developed as a long-term agent for grasshopper control in the USA [9] and in different areas of Argentina [10], [11].

Microsporidia are obligate, intracellular pathogens that are usually considered to be a unique eukaryotic phylum (Microspora) and placed in the kingdom Protista [12]. However, molecular phylogenetic analyses have revealed that they are highly derived fungi [13], [14]. Microsporidia infect almost all groups of vertebrate and invertebrate taxa [15] and have been mostly found in insects [16]. The members of the genus *Nosema* are often considered to be the most significant and widely distributed group of microsporidia, and they mainly infect Lepidoptera [17]. This genus commonly occurs in the natural populations of DBM [18]–[20] and the spores of this parasite can be ingested while the larvae feed on contaminated plant leaves [21]. Idris and Grafius [22] showed that horizontal transmission could occur through all body excretions and exuviae that contain *Nosema* spores. After ingestion, *Nosema* sp. generally infects their hosts via polar filament extrusion into the epithelial cells of the midgut. Once an insect is infected, the spores spread from cell to cell in the midgut and other tissues such as the fat body, Malpighian tubules and reproductive tissues depending on the microsporidian and host species [23]–[25]. Infective spores from midgut infections are released by cell lysis and passed into the lumen of the gut to the environment through the feces and silk of the infected hosts. The spores can also be released when an infected host dies [26].

The microsporidium *Vairimorpha imperfecta* (*Nosema bombycis* Negali) [27] has been reported to cause a major problem in the rearing of DBM and its parasitoids in the laboratory [28]. The effects of biopesticides on beneficial and non-target insects are generally assessed by measuring their acute (mortality) and sublethal toxicities [6], [29]. Sublethal physiological effects of biopesticides, such as elongation of developmental period reduction in the number of eggs produced per female, and changes in survival patterns, are considered important information to ensure safeguarding of non-target organisms, including beneficial insects, such as natural enemies [5]. The prevalence of microsporidial diseases in the host population has a negative impact on parasitoid population dynamics in the field such as *Muscidifurax raptor* (Hymenoptera: Pteromalidae), *Trichogramma nubilale* (Hymenoptera: Trichogrammatidae) and *Macrocentrus grandii* (Hymenoptera: Braconidae) [30]–[34]. However, the impact of microsporidial diseases on DBM larval parasitoids was not studied.

*Cotesia vestalis* (Haliday) [ = *C. plutellae* (Kurdjumov)] (Hymenoptera: Braconidae) is a specialist primary solitary larval endoparasitoid that attacks the first and second instars of DBM [35]. *Cotesia vestalis* is found in many regions including Europe, Asia, North America, the Caribbean and Australia [36]. However, it is more prevalent in warm climates, especially in the lowland areas of the tropics [37]–[39]. It is one of the important biological control agents commonly used in combination with other methods in integrated DBM management programs [2]. This endoparasitoid not only attacks DBM at a high rate [2], [40] but also successfully reduces its feeding damage by killing the most damaging larval stage of the pest, the fourth instar.

In this study we investigated the effects of *Nosema*-infected DBM on: (1) the parasitism rate by *C*. *vestalis* and its F2 progeny and (2) the body size, fecundity, and morphometric characteristics of *C*. *vestalis* that developed on *Nosema*-infected hosts.

## Materials and Methods

### Plant material

Cabbage plants (*Brassica oleraceae* var. *capitata*) were regularly grown to the six- to seven-leaf growth stage in plastic pots containing a mixture of sandy loam soil and peat moss (4∶1) in screen houses at the National University of Malaysia (UKM). No pesticides were applied throughout the entire study period.

### Insect sources

Healthy DBM larvae of the University Putra Malaysia strain were obtained from the Malaysian Agricultural Research and Development Institute (MARDI). This strain originated from crucifer crops in Serdang, Malaysia. It has been reared for several generations on an artificial diet in the insectary at MARDI. The colony is regularly examined microscopically to confirm that it is free of microsporidian infection. The larvae were brought to the UKM parasitology laboratory and reared on potted cabbage (*B. oleracea* var. *capitata*) in screen cages (38 cm×26 cm×26 cm) as stock culture maintained at 25±1°C, 12 h∶12 h (light:dark) photoperiod, and 45% to 65% relative humidity. Cotton wool soaked in 10% honey solution was offered to adults as food.

Cocoons (pupae) of *C. vestalis* were obtained from MARDI, Cameron Highlands, Pahang, Malaysia. The parasitoids were reared at the UKM parasitology laboratory by using DBM larvae as hosts and cotton wool soaked in 10% aqueous honey solution as a food source for the adults. For oviposition, 200 second instar DBM larvae were introduced on a potted cabbage in a wooden cage (38 cm×26 cm×26 cm) covered with a fine cloth mesh. The larvae were allowed to feed on cabbage leaves for 1 h in order to produce damage that would attract the parasitoids. A total of 15 mated female parasitoids were released into the cage and left to oviposit for 2 d. Cabbage plants were replaced in the cages as needed for larval development until pupation. Cocoons of *C. vestalis* were collected and kept individually in glass vials until adult emergence. After emergence, each female was caged (15 cm×15 cm×8 cm) with a few males until all females were mated and used in the experiment. DBM and *C. vestalis* cultures were maintained in the laboratory at 25±1°C, 45% to 65% RH, and 12 h∶12 h (light:dark) photoperiod.

### Microsporidia

*Nosema* sp. spore suspensions were harvested from naturally infected DBM collected regularly from cabbage fields in the area of Cameron Highlands, Pahang. No special permits were required for field collection and sample processing. Collection permission was obtained from the land owners. The field studies did not involve endangered or protected species. DBM were ground, purified, and then centrifuged as described in our previous study [41]. The spores were counted using a haemocytometer under a light microscope with 40× magnification following the Cantwell formula [42]. Spore suspensions ranging from 1×10^2^ to 1×10^5^ spores/µL were prepared by diluting the original spore suspensions with distilled water before storage at 4°C until further use.

### Exposure of DBM larvae to *Nosema* infection

One microliter of *Nosema* sp. spore suspension at concentrations (treatments) of 1×10^2^, 1×10^3^, 1×10^4^, and 1×10^5^ spores/µL were spread evenly on the surface of leaf discs (5 mm diameter) of the rape plant *Brassica juncea* by using the bulb end of a Pasteur pipette. Sterile water was used as a control. Second instar DBM larvae were selected randomly from uninfected colonies and placed into wells of 24-cell plastic culture plates. Each well contained one larva and one leaf disc. The control and treated larvae were kept separately under laboratory conditions mentioned above. After 24 h, 150 second instar larvae were selected from each treatment (concentration) for use in the next bioassay.

### Effects of *Nosema*-infected DBM larvae on parasitism by *C. vestalis*

The second instar DBM larvae selected for use in bioassays were randomly placed in wooden cages (30 cm×30 cm×30 cm) containing potted cabbage plants. Each cage contained 30 second instar larvae and there were five cages for each concentration of *Nosema* sp. A single mated female parasitoid was introduced into each cage for parasitism and then removed from the cages after 24 h. The DBM larvae were allowed to develop on the cabbage until pupation (cocoon formation). Cabbage plants were added as needed. Before pupation, the larvae emerge from the host and then spin a silky cocoon near the remains of the host. The parasitoid cocoons were collected using forceps, weighed using a digital electronic balance within 24 h and kept in clean ventilated plastic containers until adult emergence. The number of cocoons, non-parasitized larvae (forming DBM pupae), and emerged adult parasitoids for each concentration of *Nosema* sp. were recorded.

*Cotesia vestalis* adults (F1) that emerged from infected and uninfected host larvae in the previous experiment were used to parasitize uninfected DBM larvae to determine the effect of *Nosema* sp. on the second generation (F2) of *C*. *vestalis*. One mated F1 female *C*. *vestalis* from each treatment was randomly selected and introduced into a cage containing a cabbage plant and 30 uninfected second instar DBM larvae. Each cage considers a replication and there were three replications for each treatment. Female *C*. *vestalis* adults were removed from the cages after 24 h, and new plants were added until cocoons formed or larvae pupated. Data were recorded as described for the first generation.

### Effects of *Nosema*-infected DBM larvae on *C. vestalis* juvenile development

Only a single spore concentration was used in these experiments. The second instar larvae of DBM were fed with cabbage discs contaminated with 10^3^ spores/µL as previously described. Control larvae were fed cabbage sprayed with distilled water. After 24 h, 50 second instar larvae of DBM from each treatment were divided into 10 groups of five individuals, and then placed in ventilated plastic containers (28 cm×19 cm×11 cm) with cabbage leaves. One mated female parasitoid was introduced into each of the containers through a small hole in the container’s lid. After insertion of the parasitoid, a cotton pad soaked in honey solution was inserted through the hole as a food source for the parasitoid. Each arena contained a ratio of one female parasitoid to five DBM larvae to ensure that the majority of the hosts were parasitized. The containers were maintained under the laboratory conditions. On the basis of previous observations, we removed the parasitoids 4 h after their introduction in order to avoid superparasitism. Each larva from all treatments was individually placed in a separate 100 mL plastic container lined with moist filter paper and a fresh cabbage leaf as a food source for host larval development. The larvae were maintained under the same laboratory conditions. Leaves were replaced with fresh ones as necessary until parasitoid pupation. Cocoons were then removed, placed individually in 300 mL clean plastic containers and subsequently monitored for adult emergence. Cocoons were inspected twice daily until adult emergence (F1). The adult stage was considered to have begun when the adult completely left the cocoon. The following parameters were recorded: number of days from oviposition until the larvae egress from their host (duration of egg to larva stage) and the number of days from the cocoon appearance until adult emergence (duration of pupa stage). Adult female parasitoids that emerged from experimental DBM were kept with males that developed in healthy hosts for 24 h for mating. Mated females were then provided with uninfected second instar DBM larvae to assess the effect of *Nosema* sp. on the development of second-generation (F2) *C. vestalis* by using the same experimental procedure as described for the F1 generation.

### Effects of *Nosema*-infected DBM larvae on *C. vestalis* fecundity, and egg and body size

Ten *C. vestalis* females (1-day-old) from each of the F1 and F2 generations which had previously been treated with 10^3^ spores/µL were selected randomly and killed by freezing for 30 min. Each female was placed on a glass microscope slide with 10% saline solution (NaCl 0.85%). The abdomen was opened using a pair of #0 insect micropins to expose the ovaries under a dissecting microscope (Olympus, Tokyo, Japan). A glass coverslip was placed over the ovaries, and the mature eggs were counted (fecundity) using a stereomicroscope equipped with a lens (SMZ1500; Nikon, Japan) and a camera (Digital Sight DS-5M; Nikon). Mature eggs are transparent and spindle-shaped with a narrow pedicel at one end whereas immature eggs are smaller, opaque and barely discernible in the distal portions of the ovariole. A total of 25 eggs from each treatment were randomly selected and their length and width were measured.

Body size was assessed by measuring the hind tibia by using an ocular micrometer mounted on a dissecting microscope at 20× magnification (Leica Microsystems, Bannockburn, IL, USA). The length of the hind tibia has been used as an indicator of the body size of other parasitoids [43]. Ten F1 individuals (males and females) from the infected and uninfected groups were deep-frozen on the day of emergence and placed on microscope slides within a droplet of saline solution. The hind tibia length was measured. The body length from the top of the head to the tip of the abdomen, the wing length (distance measured between thoracic attachment points to distal tip of a detached wing), the antenna length, and ovipositor length were also measured.

### Statistical Analysis

Data were tested for normality by using the Anderson- Darling test. Transformation of data was not needed because the variances were normal and homogeneous. Parasitism rates were calculated as [% parasitism = (number of *C. vestalis* cocoons/total numbers of *C. vestalis* cocoons + *P. xylostella* pupae×100]. Regression analysis was used to evaluate any correlation between parasitism rates and *Nosema* sp. dose. Parasitism rates, percent emergence and cocoon weight were analyzed using one-way analysis of variance (ANOVA), and differences between treatment means were separated using Tukey’s test at a 5% level of significance. The statistical analyses were conducted using MiniTab software version 16. The difference between untreated and treated parasitoid means for developmental time, fecundity, and morphometrics was determined on the basis of *t*-test (*P<*0.05).

## Results

### Effects of *Nosema* sp. on parasitism, cocoon weight and adult emergence of *C. vestalis*

Percentage parasitism differed significantly across different treatments (*F* = 12.6; *df* = 4, 20; *P*<0.05, Table 1). Parasitism of controls was significantly higher than that of larvae treated with *Nosema* sp. Parasitism of *C. vestalis* on DBM larvae infected with *Nosema* sp. decreased with increasing spore dose. As expected, there was a significant negative correlation between parasitism of *Nosema*-infected DBM larvae by *C. vestalis* and spore dose for both F1 and F2 generations (F1: r^2^ = −0.81, F_(1,23)_ = 44.03, *P*<0.05; F2: r^2^ = −0.80, F_(1,13)_ = 24.71, *P*<0.05).

**Table 1:** Mean % parasitism, cocoons number and weight, number of adults and percentage of emergence of F1 *C. vestalis* developed within uninfected and *Nosema*-infected DBM larvae.

Dosage(Spores/µl)	Parasitism (%)(mean ± SE)	Dead DBMlarvae ± SE	Cocoons± SE	Cocoon weight(mg) ± SE	Adults± SE	Emergence (%)± SE
Control	90.33±1.75 a	3.6±1.40 a	23.8±1.2 a	2.25±0.1 a	21±1.6 a	87.7±3.4 a
1**×**10^2^	61.93±4.05 b	10±1.41 b	12.4±1.2 b	1.62±0.1 ab	8.8±0.4 b	73.5±7.1 ab
1**×**10^3^	58.30±3.41 b	12±1.41 bc	10.6±0.7 bc	1.86±0.4 ab	7±0.7 bc	67.1±4.6 b
1**×**10^4^	45.57±2.53 bc	12.2±0.6 bc	8.4±0.7 cd	1.63±0.1 ab	5.2±0.4 cd	62.7±4.3 bc
1**×**10^5^	36.7±11.3 c	13.8±0.4 c	7±1.1 d	1.50±0.1 b	3.6±0.7 d	49.5±6.3 c

Significantly fewer *C. vestalis* pupae (*F* = 43.5; *df* = 4, 20; *P*<0.05) and adults (*F* = 60.7; *df* = 4, 20; *P*<0.05) developed from the host larvae fed each concentration of *Nosema* sp. spores than those developed from uninfected DBM larvae. *Cotesia vestalis* cocoons obtained from the infected had lower weight (*F* = 2.68; *df* = 4,120; *P*<0.05) than those obtained from the uninfected larvae.

*Nosema* sp. had a significantly negative effect on the percentage of adult emergence (*F* = 6.98; *df* = 4, 20; *P*<0.05) at all concentrations tested (Table 1). The effect was more pronounced from host larvae fed 1×10^5^ spores/µL because less than 50% of the adults emerged from the cocoons compared with (87.7%) those that emerged developed from uninfected larvae.

The effects of *Nosema* sp. on the F2 generation of *C*. *vestalis* produced from F1 females that developed in the infected hosts showed a similar trend as those observed in the F1 generation (Table 2). Significant differences in parasitism (*F* = 6.13; *df* = 4, 10; *P*<0.05), cocoon weight (*F* = 11.12; *df* = 4,120; *P*<0.05), and percent of adult emergence (*F* = 5.8; *df* = 4, 10; *P*<0.05) were noted between the *Nosema*-infected treatments and the control (Table 2).

**Table 2:** Mean % parasitism, cocoons number and weight, number of adults and percentage of emergence of F2 *C. vestalis* developed within uninfected DBM larvae.

Dosage (Spores/µl)	Parasitism (%)(mean ± SE)	Dead DBMlarvae ± SE	Cocoons± SE	Cocoons weight(mg) ± SE	Adults ± SE	Emergence (%)± SE
Control	87.98±6.07 a	5.3±0.8 a	21.7±1.5 a	1.82±0.05 a	19±0.5 a	87.9±4.5 a
1**×**10^2^	70.67±4.17 ab	12.6±1.8 b	12.4±1.8 b	1.58±0.06 ab	8.4±0.3 b	70.6±10.3 ab
1**×**10^3^	67.77±3.93 ab	15.6±1.7 bc	9.7±1.2 bc	1.36±0.05 bc	6.3±1.5 bc	63.8±7.3 b
1**×**10^4^	44.3±13.1 b	13.3±2.9 b	6.7±1.5 cd	1.28±0.09 c	4±0.5 cd	62.5±6.2 bc
1**×**10^5^	44.04±6.98 b	20.6±1.4 c	4±0.5 d	1.24±±0.08 c	1.7±0.3 d	41.1±4.8 c

### Effects of *Nosema*-infected DBM larvae on *C. vestalis* juvenile development

**Figure 1:**
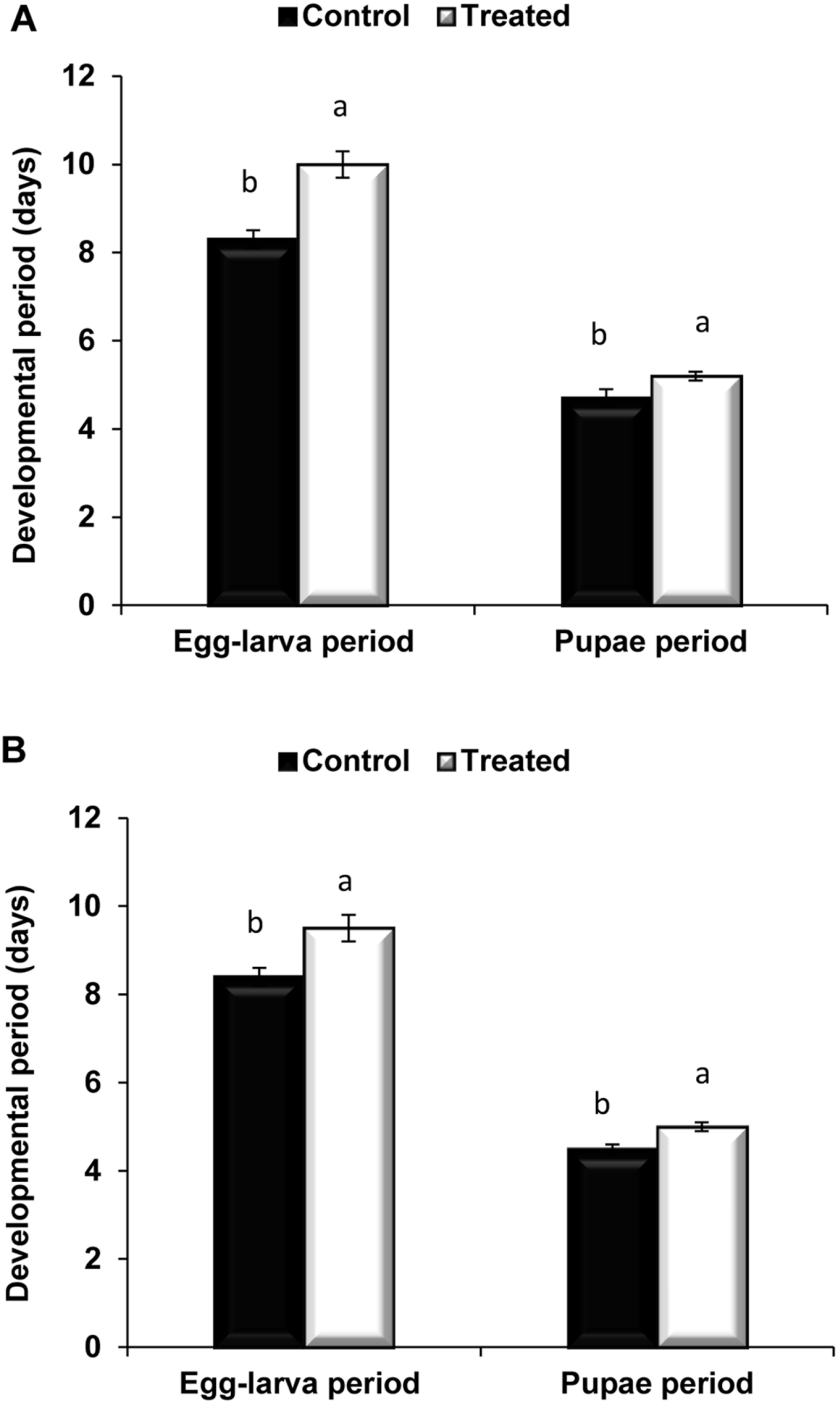
Developmental time of *C. vestalis* reared on *Nosema*-infected DBM. (**A**) Means (± SE) development time (days) of egg-larvae, pupae of F1 *C. vestalis* on uninfected and *Nosema*-infected *P. xylostella* larvae. (B) Means (± SE) development time (days) of egg-larvae, pupae of F2 *C. vestalis* on uninfected *Pllalute xylostella* larvae. Different letters above error bars indicate significant difference (Student’s t-test, *P*<0.05).

In general, the development time of different stages of *C. vestalis* from the *Nosema-*infected DBM larvae was significantly prolonged (*t* = −4.31, *P*<0.05, *df* = 21 for the egg to larval period and *t* = −2.46, *P*<0.05, *df* = 28 for the pupal period) compared with that of different stages of *C. vestalis* from the healthy DBM larvae (Figure 1A).

Similar to the effect of *Nosema* on the development time of the F1 generation, the F2 generation of *C*. *vestalis* originating from F1 females was longer in their larval (Figure 1, *t* = −2.93; *df* = 26; *P*<0.05) and pupal stages (*t* = −2.17; *df* = 27, *P*<0.05) than those of their non-treated counterparts (Figure 1B).

### Effects of *Nosema*-infected DBM larvae on *C. vestalis* fecundity and egg and body size

**Figure 2:**
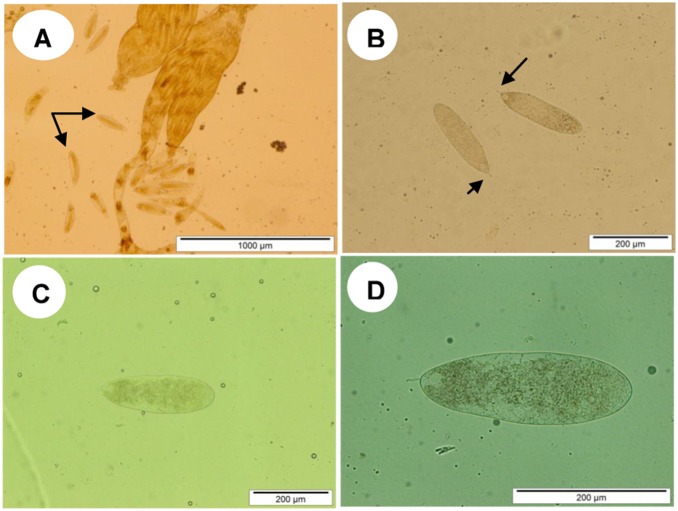
Egg stage of *C. vestalis* devolved from control and infected DBM. (A) Eggs dissected out of *C* ***.*** *vestalis* female. (B) Close-up of a typical egg (control) showing the peduncle at the front end of the egg (C) an egg from F1 *C. vestalis* (B) an egg from F2 *C. vestalis.* Magnification 40×.

**Figure 3:**
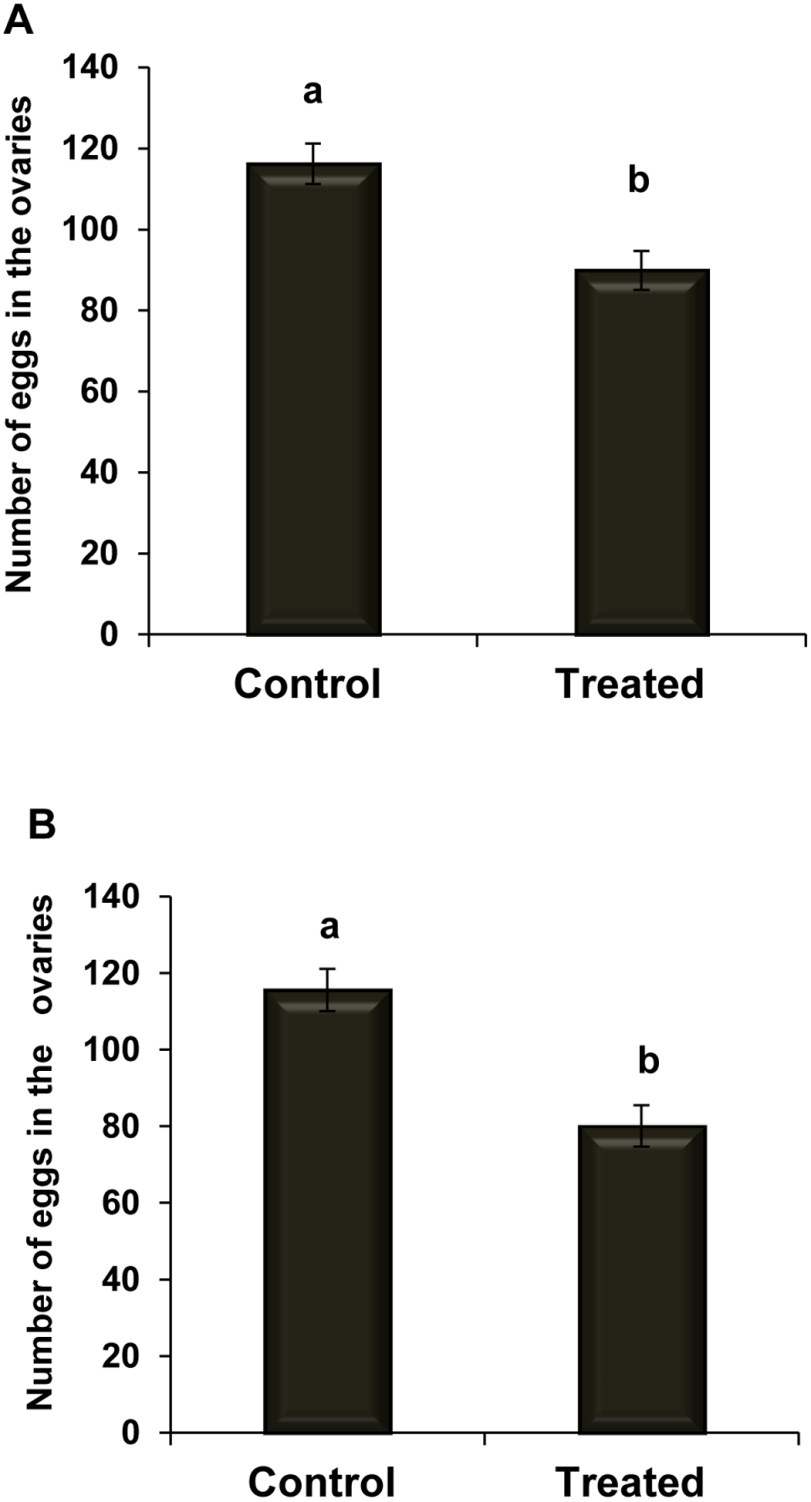
Egg production by control and *Nosema*-infected *C. vestalis*. Mean (±SE) number of eggs produced by *C*. *vestalis* females (F1) emerged from infected DBM larvae (A) and infected *C*. *vestalis* females (F2) emerged from healthy DBM larvae (B). Different letters above error bars indicate significant difference (Student’s t-test, *P*<0.05).

Adult female *C. vestalis* have two ovaries (Figure 2A); each ovary comprises several ovarioles in which the eggs develop. The eggs are spindle shaped and transparent, with a narrow peduncle at the front end (Figure 2B). As shown in Figure (3A), the number of ovarian eggs was lower (89.9±4.8) in females reared from infected DBM larvae than in those reared from healthy ones (116.2±5, *t* = 3.83, *df* = 17; *P*<0.05). As shown in Figure (3B), the mean number of eggs produced by F2 females was significantly lower (80±5.4) than those produced by the controls (115.6±5.5, *t* = 4.65, *df* = 17; *P*<0.05).

**Figure 4:**
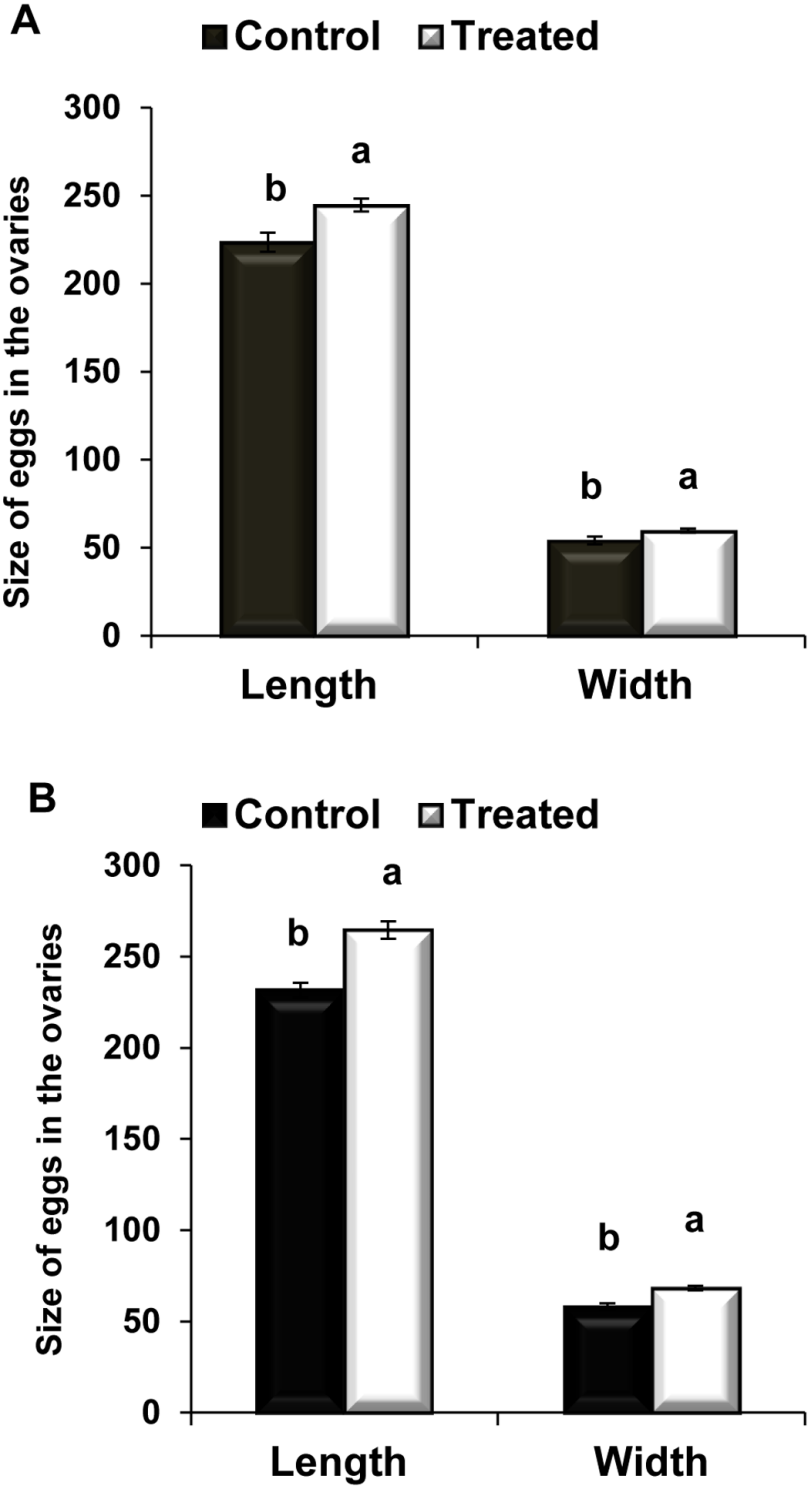
Effect of *Nosema* infection on the *C. vestalis* egg size. Mean (±) SE egg size produced by *C*. *vestalis* females (F1) emerged from infected DBM larvae (A) and infected *C*. *vestalis* females (F2) emerged from healthy DBM larvae (B). Different letters above error bars indicate significant difference (Student’s t-test, *P*<0.05).

The F1 parasitoids from treated hosts had significantly larger eggs (Figures 2C and 4A) (length: *t* = −3.22; *df* = 42; *P*<0.05; and width: *t* = −2.27; *df* = 36; *P*<0.05) than the controls. Similarly, the F2 parasitoids had larger eggs (Figures 2D and 4B) than the controls (length: *t* = −5.27; *df* = 46, *P*<0.05 and width: *t* = −4.77; *df* = 45; *P*<0.05).

The parasitoids that developed in DBM larvae infected with 10^3^ spores/µL *Nosema* sp. had significantly lower forewing length (*t* = 6, *df* = 19; *P*<0.05), antennal length (*t* = 3.1, *df* = 25; *P*<0.05), body size as estimated by hind tibial length (*t* = 2.6, *df* = 25; *P*<0.05), body length (*t* = 3.5, *df* = 27; *P*<0.05), and ovipositor length (*t* = 2.6, *df* = 25; *P*<0.05) compared to those of the controls (Table 3).

**Table 3:** Morphometric (mean) characteristics of *Cotesia vestalis* developed within uninfected and *Nosema*-infected *P. xylostella* larvae.

Morphometric characteristics (mm)	Untreated (mean ± SE)	Treated (mean ± SE)
**Forewing length**	2.34±0.02 a	2.02±0.05 b
**Hind tibia length**	0.78±0 a	0.71±0.01 b
**Antenna length**	2.59±0.05 a	2.32±0.07 b
**Body length**	2.91±0.06 a	2.58±0.06 b
**Ovipositor length**	0.29±0.02 a	0.24±0.01 b

## Discussion

Parasitism rates of *Nosema-*infected DBM larvae by *C. vestalis* were significantly reduced. The infection also caused a significant decrease in the survival of this braconid parasitoid. As expected, the highest parasitism rates were obtained from the control treatment (90.3% in F1 and 87.9% in F2), followed by those of DBM larvae treated with 10^2^ spores/µL (61.9% in F1 and 70.6% in F2). In general, the adult emergence rates of *C. vestalis* from uninfected DBM larvae were significantly higher than those of *C. vestalis* from infected DBM larvae. Parasitoids develop inside the host body, and microsporidial spores invade the host tissues such as the gut, fat body, and Malpighian tubules [44]. Thus, *Nosema* sp. is likely to invade the immature parasitoid body as well. *Nosema* spores can adversely affect the physiological processes of the developing parasitoids, resulting in the formation of small cocoons or inhibition of cocoon formation. Even, if cocoons are formed, the parasitoids might fail to emerge as adults. Similar results were reported for *Glyptapanteles liparidis*, *Microplitis tristis*, and *Tachinaephagus zealandicus* [45]–[47]. Such results can be attributed to the lack of nutritional reserves in infected parasitoids that are required for chewing their way out of the cocoon [47]. The last study reported 60% emergence failure among infected parasitoids compared with 44% emergence failure among uninfected parasitoids. Increasing *Nosema* concentration can decrease pest populations, but it can also adversely affect the parasitism rates. In the present study, the lowest parasitism rate was reported when 10^4^ and 10^5^ spores/µL concentrations were used. Some studies also reported that the increased mortality of parasitized *Diatraea saccharalis* larvae is directly proportional to increased dosage of *Nosema* spores and that high dosages produce heavy infections that prevent the parasitoid *Cotesia flavipes* from completing their development cycle [48].

Many studies on host–parasitoid–microsporidia interactions indicated that parasitoids are affected adversely by the microsporidia of their hosts [49]. In the present study, *Nosema* sp. infection of DBM had negative effects on *C. vestalis* that developed within them. For example, the egg to larval development time was prolonged up to 2 d in the F1 generation and 1 d in the F2 generation of infected parasitoids. DBM infection also prolonged the pupation period of both the generations. This result is in agreement with those reported for *Glyptapanteles liparidis* (Bouche) (Hymenoptera: Braconidae) and *Meteorus gyrator* (Hymenoptera: Braconidae) [45], [50]. However, no association between host microsporidium infection and parasitoid developmental time was observed for parasitoids from the families Encyrtidae and Pteromalidae [47], [51].

Our results showed that *Nosema* infections affect the fitness of *C. vestalis* by reducing fecundity, adult size, and other adult morphometric characteristics. Similar effects have been reported for other parasitoids [48], [52]–[55]. Previous studies have described chronic disease in the adult parasitoid *Muscidifurax raptor* (Hymenoptera: Pteromalidae) after the invasion of *Nosema muscidifuracis* in the midgut epithelium, Malpighian tubules, ovaries, and fat body of both larval and adult parasitoids [56], [57]. These studies also reported reduced fecundity in the parasitoids [58]. Lipids are the main energy resource for parasitoids and they play a key role in both survival and reproduction [59]. Many hymenopteran parasitoid species have been reported to be unable to accumulate additional lipids as adults [60], [61] due to their lack of de novo lipid synthesis from dietary sugars [62]. Therefore, adult fecundity is completely dependent on the quantity of lipids they acquire from their host during the larval stage [60], [61], [63]. As stated earlier, invading spores severely damage the fat body of the larvae [64], consequently disturbing host fat metabolism. This limits the amount of lipids available for egg production and affects various physiological functions. Changes in the levels of the analyzed carbohydrates and fatty acids in *Vairimorpha-*infected *Lymantria dispar* (Lepidoptera: Lymantridae) larvae were considerably severe to render them nutritionally unfavorable for the development of *G. Liparidis* [65]. This phenomenon might partially explain the adverse effects of *Nosema* infection of host larvae on *C. vestalis*.

*Cotesia vestalis* progeny perform better when supplied with abundant food. However, immature *C. vestalis* progeny feeding on infected DBM larvae need to complete their development within an unhealthy host and might therefore be unable to acquire the same amount of nutrients as those that could be obtained from a healthy host. The reduced quantity of available nutrients significantly affects their weight, size, and body length. Microsporidia developing within the hosts can also infect parasitoids developing within the same host [66]–[69]. Such parasitoid infection has been used to explain the detrimental effects on parasitoids caused by developing in infected hosts. Consequently, parasitoids can attain spores while they are consuming the infected host tissues and might then transmit the spores from the mother to the progeny through infected eggs. Interestingly, *C. vestalis* egg size significantly increased in the parasitoids (F1) that developed within infected DBM larvae and in infected parasitoids (F2) that developed within uninfected DBM larvae, compared to those of the controls. This increase in egg size might be caused by the changes in the reproductive physiology of the infected parasitoid. Further histological studies on parasitoid tissues are necessary to elucidate the pathway of spores inside a parasitoid’s body and ascertain whether the observed increase in egg size due to the spores or some other physiological disorder that resulted from *Nosema* infection.

When *Nosema-*infected *C*. *vestalis* parasitize the uninfected DBM larvae, the spores might be transmitted to the DBM larvae through the parasitoid eggs (vertical) or through the contaminated ovipositor or female’s body. Female parasitoids have a stinging apparatus that is used to inject maternally derived secretions (venom) into the hemocoel of their hosts at oviposition [70], [71]. This stinging apparatus might also be contaminated with spores and might therefore act as a source of infection. The number of spores continues to increase as the *C*. *vestalis* larvae develop to subsequent instars, and the spores infect different body tissues. The spores eventually infect reproductive tissues and are passed on to the next generation. This phenomenon might explain the deleterious effects of *Nosema* sp. infection on the parasitism, fecundity, and morphometric characteristics of the parasitoid F2 generation. Vertical transmission of microsporidia from mother to progeny and the possibility of its transmission via a contaminated ovipositor from infected to uninfected hosts in other insects have been discussed in detail [72]–[74]. Our previous study [41] showed that *Nosema* sp. effectively suppresses DBM populations in the laboratory. The current study revealed that *C. vestalis* on its own kill more DBM compared with combined with microsporidia. Sublethal effects on *C*. *vestalis* occur when DBM larvae are infected with *Nosema* sp. Such effects might eventually lead to parasitoid population collapse. So, it seems that it isn’t useful to apply *Nosema* as an extra control method when *C*. *vestalis* is already present.

The use of parasitoids and *Nosema* sp. to control DBM might not be effective in IPM strategies. Assessment of the risk to non-target organisms, such as pollinators and predators, is needed in order to further understand the interspecific and intergeneration *Nosema* sp. transmission mechanisms. Detailed studies are also required because DBM populations in the field might be naturally infected by a variety of other pathogens (i.e., viruses, bacteria, and fungi) or artificially affected by pesticides and/or genetically modified-derived toxins.
